# INFLUENCE OF SPLENIC IMPLANTS IN THE SUBCUTANEOUS TISSUE ON ASPLENIC ANIMALS SURVIVAL

**DOI:** 10.1590/0102-672020180001e1364

**Published:** 2018-07-02

**Authors:** Renan Kleber Costa TEIXEIRA, Laryssa de Aquino SANTIAGO, Yan de Assis SASAKI, Vitor Nagai YAMAKI, Daniel Haber FEIJÓ, Marcus Vinicius Henriques BRITO, Edson Yuzur YASOJIMA, Andy PETROIANU

**Affiliations:** 1 Laboratório de Cirurgia Experimental da Faculdade de Medicina da Universidade Estadual do Pará - UEPA, Belém, PA; 2 Universidade Federal de Minas Gerais - UFMG, Minas Gerais, MG, Brazil

**Keywords:** Spleen, Splenectomy, Rats, Transplants, Baço, Esplenectomia, Transplante autólogo, Rato.

## Abstract

***Background*:**

The best site for splenic implant was not defined, mainly evaluating the functionality of the implant.

***Aim*:**

To evaluate the effects of autogenous splenic implantation on the subcutaneous tissue in the survival of splenectomized rats.

***Method*:**

Twenty-one randomly assigned rats were studied in three groups (n=7): group 1 - manipulation of the abdominal cavity and preservation of the spleen; group 2 - total splenectomy; group 3 - splenectomy and implant of the tissue removed in the subcutaneous. The animals were followed for 90 days postoperatively.

***Results*:**

There was a higher mortality in groups 2 (p=0.0072) and 3 (p=0.0172) in relation to group 1. There was no difference between groups 2 and 3 (p=0.9817).

***Conclusion*:**

The splenic implant in the subcutaneous is ineffective in the survival of rats submitted to splenectomy.

## RESUMO

***Racional*:**

O melhor sítio para implante esplênico não foi definido, principalmente avaliando a funcionalidade do implante.

***Objetivo*:**

Avaliar os efeitos do implante esplênico autógeno subcutâneo na sobrevida de ratos esplenectomizados.

**Métodos:**

Foram estudados 21 ratos alocados aleatoriamente em três grupos (n=7): grupo 1 - manipulação da cavidade abdominal e preservação do baço; grupo 2 - esplenectomia total; grupo 3 - esplenectomia e implante do tecido retirado no subcutâneo. Os animais foram acompanhados por 90 dias pós-operatórios.

**Resultados:**

Houve mortalidade maior nos grupos 2 (p=0,0072) e 3 (p=0,0172) em relação ao grupo 1. Não houve diferença entre os grupos 2 e 3 (p=0,9817).

**Conclusão:**

O implante esplênico no subcutâneo é ineficaz na sobrevida de ratos submetidos à esplenectomia.

## INTRODUCTION

The spleen was considered until a few decades ago as a superfluous organ, whose removal does not alter organic homeostasis nor cause complications^9^ ^,^ ^17^. However, judicious studies have shown many adversities due to splenectomy, mainly with immunosuppression, severe sepsis, disorders of lipid metabolism, functional alterations of the liver and bone marrow^3^ ^,^ ^8^ ^,^ ^9^ ^,^ ^17^ ^,^ ^18^. The most serious event in asplenic patients is fulminant sepsis and early mortality in relation to people with spleen^3^ ^,^ ^9^ ^,^ ^18^. 

To prevent the complications of asplenia, the current trend in trauma, oncology and hematology has been treatment with preservation of the spleen^1^ ^,^ ^2^ ^,^ ^4^. If splenic surgery is required, conservative operations of part of the spleen or, where this is not possible, splenic implants. 

According to the literature, the splenic implant to maintain its function, should be done in the abdomen, in a venous drainage system for the portal system ^4^ ^,^ ^5^, thus carrying the splenic products to the liver, which will complete the metabolism initiated by the spleen or the organic defense. Another relevant aspect is the amount of tissue implanted that should be greater than 25% of a normal spleen, in order to avoid splenic insufficiency^7^.

Although complications resulting from asplenia have been found and are avoided with implants, it has not been determined whether the implant site interferes with mortality. 

The aim of this study was to evaluate whether the autogenous splenic implant performed in the subcutaneous tissue interferes with the survival of splenectomized rats.

## METHODS

This study was performed on 21 male Wistar rats (*Rattus norvegicus*), 15-20 weeks old and weighing between 250-300 g. They were kept in the Experimental Surgery Laboratory of the State University of Pará, Belém, PA in a controlled environment. Water and feed were offered without limit. This research followed the standards for animal experimentation (Law 11.794 / 08) and the project was approved by the Ethics Committee of the State University of Pará, protocol 27/11.

The animals were randomly assigned into three groups (n=7): group 1 - laparotomy and intra-abdominal manipulation, with preservation of the spleen; group 2 - submitted to total splenectomy; group 3 - total splenectomy and implantation of the splenic tissue removed in the subcutaneous.

Operative procedures were performed under general anesthesia (ketamine 70 mg/kg and xylazine 10 mg/kg, intraperitoneal) and aseptic conditions. All animals were submitted to median laparotomy supra and paraumbilical with extension of 3 cm. In groups 2 and 3, the splenic vessels were ligated with 4-0 cotton thread, and then the spleen was withdrawn. In group 3, the spleen was weighed and two slices were removed, corresponding to 30% of the splenic tissue. These segments were implanted into the subcutaneous tissue and fixed with a 6-0 nylon stitch. The closure of the abdominal cavity of all the animals was in two planes: musculoapouneurotic with nylon thread 4-0 and skin with nylon 6-0.

Postoperatively, they were housed in individual cages, given analgesia (dipyrone 30 mg/kg 8/8 h) for five days, water and feed at will. The postoperative follow-up was 90 days or until death. The rats that died during the follow-up period were submitted to necropsy to identify the splenic implant and the cause of death. Those who survived for 90 days were killed with overdose of drugs used in anesthesia and underwent necropsy to identify subcutaneous implants.

### Statistical analysis

BioEstat^®^ software 5.4 (Belém, PA, Brazil) was used to perform statistical analysis. Survival analysis was assessed by the Klapan-Meier curves and compared by the Log-Rank test. A value of p<0.05 was used for significance of the comparisons between the three groups.

## RESULTS

**FIGURE 1:**
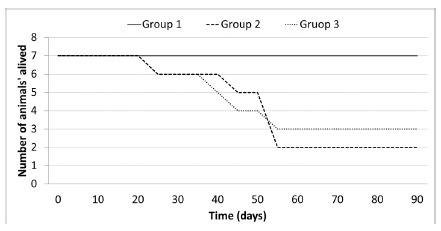
Survival curves according to the groups, 1 (with spleen), 2 (splenectomy) and 3 (splenectomy and splenic implant in the subcutaneous)

All animals in group 1 survived throughout the follow-up period. In group 2, there were five deaths, one at 30°, 41°, 50° and two at 52° postoperative days (p=0.0072) in relation to group 1. In group 3, there were four deaths at 21°, 36°, 40° and 45° postoperative days (p=0.0172) in relation to group 1. There was no difference in survival between groups 2 and 3 (p=0.9817, Figure 1). Necropsy showed that all rats died due to *E. coli* peritonitis.

All the animals presented the splenic tissue implanted intact and with vascularization in its contour.

## DISCUSSION

Conservative spleen operations^11-14^ are becoming increasingly common in many services, both in trauma and in diseases related to the spleen. The splenic implant is reserved for cases in which it is not possible to preserve part of the eutopic spleen, due to generalized ischemia or technical difficulties^1^ ^,^ ^2^ ^,^ ^4^ ^-^ ^7^ ^,^ ^15^. Implanted splenic tissue reacquires morphofunctional integrity in up to three months, since it is implanted in tissue with venous drainage to the portal vein and in sufficient quantity^5^ ^,^ ^6^ ^,^ ^15^. 

In this study, splenic implantation in the subcutaneous region was accompanied by deaths in more than half of the animals submitted to splenectomy, with no difference in relation to implanted asplenics. This result occurred despite all implants maintaining their vitality. According to the literature, the splenic tissue maintains vitality in any tissue where it is implanted^1^ ^,^ ^4^ ^,^ ^6^ ^,^ ^15^. This knowledge existed almost a century ago when studies of splenocytes, natural splenic implants, began after major trauma to the spleen. Part of this ruptured organ is attached to various parts of the abdomen (peritoneum, omentum, intestinal serosa and ligaments) and even extra-abdominal, in the chest (pleura, mediastinum, lung) and lower limbs (subcutaneous, fascia and muscles)^16^. 

In the literature there is evidence that splenic implants in the greater omentum, mesocolon and retroperitoneum increase the resistance of animals to *Escherichia coli* and *Staphilococcus aureus* infections, reducing mortality after splenectomy. However, splenic implants outside drainage tissue to the portal system, although they remain viable, do not function properly and according to this study, they are accompanied by high mortality^8^ ^,^ ^18^. Death by peritonitis showed that rats, usually very resistant to infection, when asplenic lose the ability to defend against peritoneal bacteria, even in the presence of splenic implants in the subcutaneous.

There is a need for further studies to understand the importance of drainage of the splenic products to the liver through the portal flow.

## CONCLUSION

The splenic implant in the subcutaneous was ineffective in the survival of rats submitted to splenectomy.
